# Sensitivity of Methods for Diagnosing Noise-Induced Hearing Loss in Cases of Exposures Including Intense Low-Frequency Noise

**DOI:** 10.1177/23312165241240353

**Published:** 2024-03-28

**Authors:** Brian C.J. Moore, Graham Cox

**Affiliations:** 1 Cambridge Hearing Group, 98528 Department of Psychology, University of Cambridge, Cambridge, UK; 2 ENT Department (retired), 6397 Oxford University Hospitals NHS Foundation Trust, Oxford, UK

**Keywords:** noise-induced hearing loss, low-frequency noise, diagnostic methods, armoured vehicles

## Abstract

Exposure to intense low-frequency sounds, for example inside tanks and armoured vehicles, can lead to noise-induced hearing loss (NIHL) with a variable audiometric pattern, including low- and mid-frequency hearing loss. It is not known how well existing methods for diagnosing NIHL apply in such cases. Here, the audiograms of 68 military personnel (mostly veterans) who had been exposed to intense low-frequency noise (together with other types of noise) and who had low-frequency hearing loss (defined as a pure-tone average loss at 0.25, 0.5 and 1 kHz ≥20 dB) were used to assess the sensitivity of three diagnostic methods: the method of Coles, Lutman and Buffin, denoted CLB, which depends on the identification of a notch or bulge in the audiogram near 4 kHz, and two methods specifically intended for diagnosing NIHL sustained during military service, the rM-NIHL method, which depends on the identification of a notch or bulge in the audiogram near 4 kHz and/or a hearing loss at high frequencies greater than expected from age alone, and the MLP(18) method based on a multi-layer perceptron. The proportion of individuals receiving a positive diagnosis for either or both ears, which provides an approximate measure of sensitivity, was 0.40 for the CLB method, 0.79 for the rM-NIHL method and 1.0 for the MLP(18) method. It is concluded that the MLP(18) method is suitable for diagnosing NIHL sustained during military service whether or not the exposure includes intense low-frequency sounds.

## Introduction

The audiometric configuration associated with noise-induced hearing loss (NIHL) depends on the type of noise exposure. For exposures involving steady broadband sounds, such as factory noise, NIHL is usually associated with a notch or bulge in the audiogram centred near 4 kHz (Passchier-Vermeer, 1974; Smoorenburg, 1992). Exposures involving intense impulsive sounds, such as occur during military service (Jokel et al., 2019), lead to a more variable audiometric pattern (Lowe & Moore, 2021; Moore, 2020). Sometimes such exposures also lead to a notch or bulge in the audiogram centred near 4 or 6 kHz, but sometimes they lead to a hearing loss that increases progressively with increasing frequency up to 8 kHz. The frequency at which the hearing loss is greatest varies markedly across individuals (Lowe & Moore, 2021; Moore, 2020). Exposures that involve intense low-frequency noise can lead to a low- or mid-frequency loss. Doroshenko and Stepchuk (1983), cited in Schust (2004), studied compressor operators exposed to infrasound (frequencies below 20 Hz) combined with steady noise at higher frequencies for a daily period of 6.5 h in a cross-sectional study. The control group consisted of workers exposed to industrial noise without any infrasound. The exposure duration was from 1 to 20 years. Combined infrasound and higher frequency noise exposure caused significantly greater hearing loss than higher frequency exposure alone, especially at low and medium frequencies. Another example is the hearing of police and other motorcyclists. The wind blowing round their helmets causes noise whose energy is maximal at 0.25 and 0.5 kHz. This leads to NIHL that tends to be greatest at low frequencies (McCombe et al., 1995). Several studies involving animals have shown that exposure to intense low-frequency tones or bands of noise can lead to temporary or permanent hearing loss and damage to the hair cells in the cochlea over a wide range of frequencies (Burdick et al., 1978; Harding & Bohne, 2009; Liu et al., 2022; Mills et al., 1983).

Because different types of noise exposure lead to different audiometric patterns, methods for diagnosing NIHL should be based partly on consideration of the type of exposure (Moore et al., 2022a). All diagnostic methods include the requirement that there should have been exposure to noise with the potential to cause hearing loss, and it is assumed here that this requirement has been met. Most methods for diagnosing NIHL produced by exposure to steady broadband noise assess whether there is a notch or bulge in the audiogram centred near 4 kHz (Coles et al., 2000; Niskar et al., 2001; Phillips et al., 2010; Pudrith et al., 2022). For example, the method of Coles et al. (2000), called here the CLB method, has two main requirements in terms of the audiogram: (a) a single measurement of the hearing threshold level (HTL) at 3, 4 or 6 kHz should be at least 10 dB greater than the HTL at 1 or 2 kHz, i.e. hearing loss should be greater at high than at low frequencies and (b) there should be a downward notch or bulge in the audiogram of 10 dB or more in the range 3–6 kHz, assessed relative to the HTLs that would be expected from age alone.

Several methods have been designed specifically for diagnosing NIHL sustained during military service, denoted here M-NIHL (Moore, 2020; Moore et al., 2022b; Moore & Schlittenlacher, 2023). The first two of these methods, denoted here the M-NIHL (2020) method and the rM-NIHL method, respectively, both have a requirement similar to the first requirement of the CLB method, i.e. greater hearing loss for at least one high frequency than for lower frequencies. In addition, they require the identification of either a notch in the audiogram centred near 4 or 6 kHz or a hearing loss at high frequencies that is greater than expected from age alone (referred to as excess HF), based on ISO 7029 (2017), or both. The M-NIHL (2020) method was reported to have high sensitivity (the proportion of cases with NIHL that are correctly diagnosed as having NIHL) but only moderate specificity (the proportion of cases without NIHL that are correctly diagnosed as not having NIHL) (Moore, 2020; Moore & von Gablenz, 2021). The rM-NIHL method used slightly more stringent criteria than the M-NIHL (2020) method in terms of the required notch depth or the magnitude of the excess HF, and consequently had slightly lower sensitivity than the M-NIHL (2020) method but markedly better specificity (Moore et al., 2022b). Both the M-NIHL (2020) method and the rM-NIHL method make a diagnosis separately for each ear. An individual is deemed to have M-NIHL if the diagnosis is positive for either or both ears.

A different approach to the diagnosis of M-NIHL, based on the use of multi-layer perceptrons (MLPs, a category of deep neural networks), was described by Moore and Schlittenlacher (2023). The MLPs were trained so as to classify individuals as belonging to a noise-exposed group composed of former military personnel or to a non-exposed age-matched control group, based on their audiograms and ages, thereby automatically identifying the features of the audiogram that provide optimal classification. Two databases (noise-exposed and non-noise-exposed) were used for training and validation of the MLPs and two independent databases were used for evaluation of the trained MLPs. The best-performing MLP, denoted MLP(18), used age-expected HTLs and the audiometric thresholds for each ear at 1, 2, 3, 4, 6 and 8 kHz as input features. This achieved high sensitivity (0.986), intermediate between that for the M-NIHL (2020) and rM-NIHL methods, and also achieved high specificity (0.902). The specificity was markedly higher than for the M-NIHL (2020) and rM-NIHL methods. Note that, in contrast to the M-NIHL (2020) and rM-NIHL methods, the MLP(18) method diagnoses whether or not an *individual* has M-NIHL based on age and the audiogram for both ears.

The authors are not aware of diagnostic methods for NIHL intended specifically for application to cases of exposure to intense low-frequency noise. Military personnel who work in tanks or other armoured vehicles are exposed to intense low-frequency noise, often in combination with other types of noise. Although the authors have seen cases of low- or mid-frequency hearing loss for military personnel with such exposures, reports of this pattern of hearing loss are rare. However, Lowe and Moore (2021) reported a few cases of former military personnel who had their greatest hearing loss at low or mid frequencies, and a few such cases were included in the databases used to develop the rM-NIHL and MLP(18) methods. The present paper evaluates the sensitivity of the CLB, rM-NIHL and MLP(18) methods for diagnosing M-NIHL for a population of current or former military personnel who were exposed to intense low-frequency noise and had some degree of low-frequency hearing loss. The focus was on individuals with some degree of low-frequency hearing loss, because such cases might not meet the first requirement of the CLB and rM-NIHL methods, namely that the hearing loss for at least one high frequency should be greater than the hearing loss at low frequencies, and because there appear to be no previous evaluations of the sensitivity of existing diagnostic methods for individuals with low-frequency hearing loss.

## Study Population

The data were extracted from an anonymised database of records of former military personnel. Since the study involved an analysis of pre-existing anonymised data, no ethical approval was required. The study group was derived from the audiograms of 524 individuals (1048 ears), all men, who were serving or had served in the British armed forces and all of whom had been exposed to variable amounts of intense low-frequency noise, often together with other types of noise. Records were available for some females who had been exposed to intense low-frequency noise, some of whom had low- and mid-frequency hearing loss, but the number of females was too small to allow a meaningful analysis. All of the audiograms were obtained according to the recommendations of the British Society of Audiology (2018). All of the individuals had made claims for compensation for M-NIHL sustained during their military service. All had a medical examination on entry to medical service which included a hearing assessment. All were considered to have suitable hearing for entry into military service, indicating near-normal hearing.

Medical causation for hearing loss other than noise exposure was excluded by scrutiny of the medical records and by interview. None had significant noise exposure from hobbies or recreation and none had significant unprotected noise exposure in civilian employment.

Detailed questioning about the type of noise exposure revealed considerable heterogeneity. In part this appeared to be related to the absence of any standard type of hearing protection used by members of the British armed forces. In addition, the noise exposure of those within or close to armoured vehicles varied significantly depending on their role. Some used communication headsets of different types, whilst others in the same vehicle did not. All of the individuals had also been exposed to high-intensity impact sounds from small arms fire, during basic training and during annual weapons tests.

From the group of 524 individuals, 68 were identified as having low-frequency hearing loss in one or both ears, defined as a pure-tone average (PTA) threshold across 0.25, 0.5 and 1 kHz ≥ 20 dB HL. Their average age was 46 years (standard deviation, SD = 9 years). The average time interval between the start of the exposure and the time at which the audiogram was obtained was 28 years (SD = 10 years). The average duration of the exposure was 14 years (SD = 8 years). The average time between the end of the exposure and the time at which the audiogram was obtained was 13.5 years (SD = 9 years). The majority of the 68 had considerable exposure to low-frequency noise, often with simultaneous vibration, from tanks and other armoured vehicles. The majority had served in regiments that were part of the Royal Armoured Corps. Others in the study group had served in the armoured or mechanised infantry, or were attached to regiments using armoured vehicles. These included soldiers serving in signals, engineering, and logistics regiments. A minority of the 68 had served in the Royal Air Force, Royal Navy, and Royal Marines. These had material exposure to intense low-frequency sound from helicopters, marine engines, and aircraft engines.

Of the 68 individuals, data were available for 64 about whether they had experienced: a temporary dulling of hearing and/or tinnitus soon after one or more episodes of noise exposure (denoted temporary threshold shift, TTS); whether they currently had tinnitus; and whether they currently had hyperacusis. Of those, 46 reported TTS, 60 had tinnitus now, and 38 had hyperacusis now. Forty-four had TTS combined with tinnitus now, 29 had TTS combined with hyperacusis now, and 38 had hyperacusis now combined with tinnitus now (i.e. all of those with hyperacusis also had tinnitus). Twenty-nine had TTS, tinnitus now and hyperacusis now. Of the 60 who had tinnitus now, the tinnitus of 32 fell in grades 3–4 (severe or catastrophic), using the grading scheme of McCombe et al. (2001). For 14 individuals, the hyperacusis was rated as severe or very severe.

**Figure 1.**
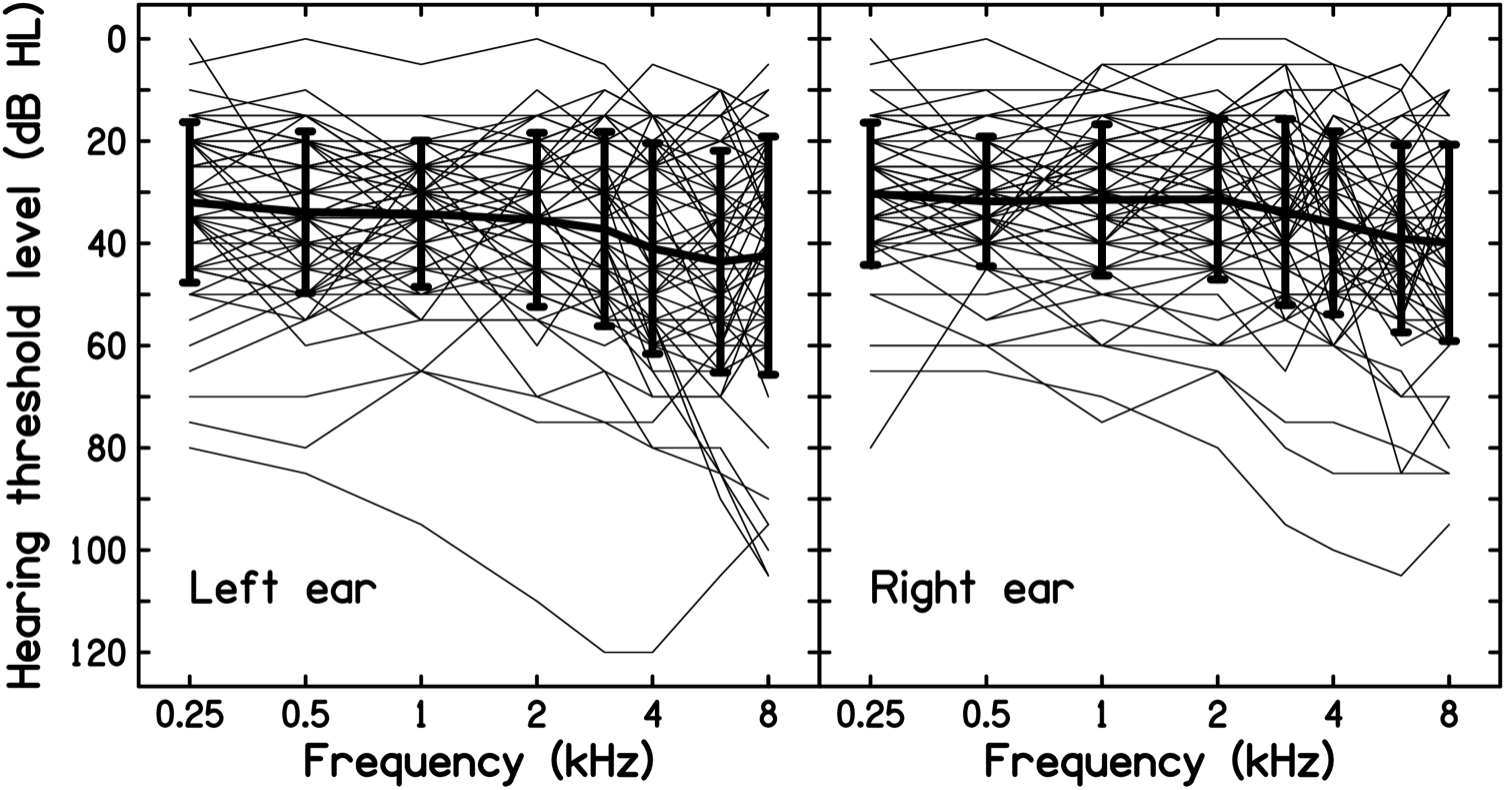
The thin traces show individual audiograms for the right and left ears for the 68 noise-exposed individuals with low-frequency hearing loss. The thick traces show the mean audiograms and the error bars show ±1 SD.

The audiograms for each ear of each individual in the study group are shown in Figure 1. It is clear that there was substantial variability in the shapes of the audiograms and in the severity of hearing loss. For both ears, the average audiogram (thick lines) was almost flat for frequencies from 0.25 to 2 kHz; the hearing loss increased slightly at higher frequencies. The average audiogram did not show a clear notch or bulge at 4 or 6 kHz for either ear. Based on the PTA threshold across 0.25, 0.5 and 1 kHz, 17 individuals showed an interaural asymmetry ≥ 10 dB, 11 with greater loss for the left ear. This may reflect asymmetry in the exposures, for example if one ear was protected but the other was left unprotected to provide situational awareness.

## Sensitivity of Three Diagnostic Methods for the Study Population

Unfortunately, there is no “gold standard” for diagnosing M-NIHL with which a diagnostic method can be compared. The best that can be done is to evaluate sensitivity using a population that is highly likely to have M-NIHL. It was assumed that the great majority of the study population here did have M-NIHL, so sensitivity estimates were based on the proportion of individuals in the study population who received a positive diagnosis of M-NIHL for either or both ears. Since some of the individuals may not have had M-NIHL, the resulting sensitivity estimates represent a lower bound. The three diagnostic methods were the CLB method (Coles et al., 2000), which has been widely used in the UK for the diagnosis of NIHL, the rM-NIHL method (Moore et al., 2022b), and the MLP(18) method (Moore & Schlittenlacher, 2023). The reader is referred to the original papers for detailed descriptions of these methods.

For the CLB method, only 27 of the 68 individuals received a positive diagnosis for either or both ears, corresponding to a sensitivity of 0.40. This low sensitivity was a consequence of the fact that most of the audiograms did not show a clear notch or a bulge at 4 kHz. Also, about 20% of the audiograms did not meet the requirement of greater hearing loss at high frequencies than at low frequencies. For the rM-NIHL method, 54 of the 68 individuals received a positive diagnosis for either or both ears, corresponding to a sensitivity of 0.79. Sensitivity was much greater for the rM-NIHL method than for the CLB method because many of the individuals did not meet the notch requirement but did show sufficient excess HF, i.e. hearing loss at high frequencies greater than expected from age alone. The negative diagnoses for the rM-NIHL method occurred mainly for cases where the audiograms did not meet the requirement of greater hearing loss at high frequencies than at low frequencies. For the MLP(18) method, all 68 individuals received a positive diagnosis, indicating a sensitivity approaching 1.

## Discussion

It is remarkable that the MLP(18) method achieved a sensitivity approaching 1, because the database of noise-exposed individuals that was used to train the MLP(18) network contained only a few individuals with low-frequency loss. These few cases may have been sufficient for training. Also, the MLP(18) method only made use of HTLs at audiometric frequencies of 1 kHz and above. This suggests that there are features of the audiogram for frequencies of 1 kHz and above which indicate a diagnosis of M-NIHL for individuals exposed to intense low-frequency noise. It is not entirely surprising that the MLP(18) method had high sensitivity despite not having information about the audiometric thresholds at 0.25 and 0.5 kHz, because the average audiometric threshold across 0.25 and 0.5 kHz was highly correlated with the audiometric threshold at 1 kHz; the correlation was 0.72 for the right ears and 0.74 for the left ears.

The MLP(18) consists of: an input layer that accepts the audiometric threshold for each ear for frequencies from 1 to 8 kHz and AAHL values derived from ISO (7029); a layer with two hidden units, and a single output unit whose value determines whether or not the diagnosis is positive. There are weights from each input unit to each hidden unit and from each hidden unit to the output unit. The higher the weight, the higher the strength of a connection. It is noteworthy that examination of the weights in the MLP(18) network showed a high weight for the connection from the audiometric threshold at 1 kHz for the left ear to hidden unit 1. Whatever the reason for the high sensitivity of the MLP(18) method, the results suggest that this method is suitable for the diagnosis of M-NIHL regardless of whether or not the exposure includes intense low-frequency noise.

Only 68 individuals from the total of 524 whose exposure included intense low-frequency noise had low-frequency hearing loss as defined here. This may reflect individual differences in the amount or type of exposure, as described earlier, or in the use and type of hearing protection. It may also reflect individual differences in susceptibility to the effects of low-frequency noise, perhaps related to differences in the transmission of low-frequency sound through the middle ear (Aibara et al., 2001; Rosowski, 1991).

As noted earlier, most of the individuals in the study sample had been exposed to intense vibration as well as noise. Several studies have shown that intense vibration can increase the deleterious effects of noise on hearing (Pettersson et al., 2012; Turcot et al., 2015), perhaps partly because vibration reduces peripheral blood flow, including the blood supply to the ear. Vibration is likely to be a contributing factor to the hearing loss of the study sample used here. Unfortunately, it is hard to protect military personnel from the vibration inside armoured vehicles, and passive noise attenuation via earplugs or earmuffs tends to be small at low frequencies (Berger, 2000; Murphy et al., 2015; Neitzel & Seixas, 2005). Hence, there is an urgent need to improve the performance of hearing protection at low frequencies, perhaps via better active noise cancellation, and to reduce the low-frequency noise and vibration inside armoured vehicles, in order to reduce the risk of damage to hearing.

This paper has emphasised the use of the audiogram in the diagnosis of M-NIHL sustained during military service. However, it is also important to obtain a full medical history to rule out possible causes of hearing loss other than noise exposure (Moore et al., 2022a), as was done here. It is also important to document the types and durations of noise exposures of the individual, again as done here, to assess whether the individual experienced a dulling of hearing or tinnitus following the exposures (Brungart et al., 2019; Grant et al., 2021), and to assess whether other symptoms associated with noise exposure, such as tinnitus and hyperacusis, are present (Lowe & Moore, 2021). A final diagnosis of NIHL for a particular individual should take account of these aspects as well as the outcome of a diagnostic test based on the audiogram.

## Limitations

Some limitations of this study should be noted. Firstly, the study sample was a not random or representative samples of former military personnel. The sample was restricted to those claiming compensation for NIHL, who had a history of exposure to low-frequency noise, and who had low-frequency hearing loss. The fact that they were claiming compensation increased the likelihood of them having M-NIHL, making them suitable for estimating the sensitivity of the three methods, but is associated with the risk that the hearing loss was exaggerated. This risk is mitigated by the fact that all of the audiograms for the study sample were obtained according to the recommendations of the British Society of Audiology (2018), which meant that the procedure incorporated measures of response consistency. Another limitation is that the reports of the individuals in the study sample about their noise exposure and their experiences of temporary dulling of hearing or tinnitus depended on their memories of events that happened many years earlier.

## Summary and Conclusions

The audiograms of 68 military personnel (mostly veterans) who had been exposed to intense low-frequency noise (together with other types of noise) and who had low-frequency hearing loss (defined as a PTA loss at 0.25, 0.5 and 1 kHz ≥20 dB) were used to assess the sensitivity of three methods for diagnosing NIHL:

1. The method of Coles et al. (2000), denoted CLB, which has been widely used in the UK for the diagnosis of NIHL in a medico-legal context.
2. The rM-NIHL method (Moore et al., 2022b), which was specifically intended for diagnosing NIHL sustained during military service.
3. The MLP(18) method based on a multi-layer perceptron (Moore & Schlittenlacher, 2023). The MLP(18) network was trained using a database of ages and audiograms of noise-exposed military personnel and a control database of non-exposed individuals, so the MLP(18) method was also specifically intended for diagnosing NIHL sustained during military service.

The proportion of individuals receiving a positive diagnosis for either or both ears, which provides a lower bound estimate of sensitivity, was 0.40 for the CLB method, 0.79 for the rM-NIHL method, and 1.0 for the MLP(18) method. It is concluded that the MLP(18) method is suitable for diagnosing M-NIHL whether or not the exposure includes intense low-frequency sounds.

## References

[bibr1-23312165241240353] Aibara R. Welsh J. T. Puria S. Goode R. L. (2001). Human middle-ear sound transfer function and cochlear input impedance. Hearing Research, 152, 100–109. 10.1016/S0378-5955(00)00240-9 11223285

[bibr2-23312165241240353] Berger E. H. (2000). Hearing protection devices. In Berger E. Royster L. Royster J. Driscoll D. Layne M. (Eds.), The noise manual (5th Ed, pp. 379–454). American Industrial Hygiene Association.

[bibr3-23312165241240353] British Society of Audiology. (2018). Recommended procedure: Pure-tone air-conduction and bone-conduction threshold audiometry with and without masking. British Society of Audiology.

[bibr4-23312165241240353] Brungart D. S. Barrett M. E. Schurman J. Sheffield B. Ramos L. Martorana R. Galloza H. (2019). Relationship between subjective reports of temporary threshold shift and the prevalence of hearing problems in military personnel. Trends in Hearing, 23, 2331216519872601. 10.1177/2331216519872601 31524086 PMC6747866

[bibr5-23312165241240353] Burdick C. K. Patterson J. H. Mozo B. T. Camp R. T. Jr . (1978). Threshold shifts in chinchillas exposed to octave bands of noise centered at 63 and 1000 Hz for three days(a). The Journal of the Acoustical Society of America, 64, 458–466. 10.1121/1.382017 712007

[bibr6-23312165241240353] Coles R. R. Lutman M. E. Buffin J. T. (2000). Guidelines on the diagnosis of noise-induced hearing loss for medicolegal purposes. Clinical Otolaryngology, 25, 264–273. 10.1046/j.1365-2273.2000.00368.x 10971532

[bibr7-23312165241240353] Doroshenko P. N. Stepchuk I. D. (1983). Health related assessment of combined effect of infrasound and low­frequency noise on the acoustic and vestibular analyser of compressor operators. Gigiena Truda I Professional'nye Zabolevaniya, 1, 35–38. 6600701

[bibr8-23312165241240353] Grant K. W. Kubli L. R. Phatak S. A. Galloza H. Brungart D. S. (2021). Estimated prevalence of functional hearing difficulties in blast-exposed service members with normal to near-normal-hearing thresholds. Ear and Hearing, 42, 1615–1626. 10.1097/AUD.0000000000001067 34108398

[bibr9-23312165241240353] Harding G. W. Bohne B. A. (2009). Relation of focal hair-cell lesions to noise-exposure parameters from a 4- or a 0.5-kHz octave band of noise. Hearing Research, 254, 54–63. 10.1016/j.heares.2009.04.011 19393307

[bibr10-23312165241240353] ISO 7029. (2017). Acoustics - Statistical distribution of hearing thresholds related to age and gender (pp. 1–22). International Organization for Standardization.

[bibr11-23312165241240353] Jokel C. Yankaskas K. Robinette M. B. (2019). Noise of military weapons, ground vehicles, planes and ships. The Journal of the Acoustical Society of America, 146, 3832–3838. 10.1121/1.5134069 31795677

[bibr12-23312165241240353] Liu J. Antisdel J. Liu C. Chen M. Dong P. Fahlman R. Ma F. Yu Y. (2022). Extensive hearing loss induced by low-frequency noise exposure. Laryngoscope Investigative Otolaryngology, 7, 564–570. 10.1002/lio2.752 35434351 PMC9008144

[bibr13-23312165241240353] Lowe D. Moore B. C. J. (2021). Audiometric assessment of hearing loss sustained during military service. The Journal of the Acoustical Society of America, 150, 1030–1043. 10.1121/10.0005846 34470327

[bibr14-23312165241240353] McCombe A. Baguley D. Coles R. McKenna L. McKinney C. Windle-Taylor P. British Association of Otolaryngologists H. Neck S. (2001). Guidelines for the grading of tinnitus severity: The results of a working group commissioned by the British association of otolaryngologists, head and neck surgeons, 1999. Clinical Otolaryngology, 26, 388–393. 10.1046/j.1365-2273.2001.00490.x 11678946

[bibr15-23312165241240353] McCombe A. W. Binnington J. Davis A. Spencer H. (1995). Hearing loss and motorcyclists. Journal of Laryngology and Otology, 109, 599–604. 10.1017/s0022215100130828 7561464

[bibr16-23312165241240353] Mills J. H. Osguthorpe J. D. Burdick C. K. Patterson J. H. Mozo B. (1983). Temporary threshold shifts produced by exposure to low-frequency noises. The Journal of the Acoustical Society of America, 73, 918–923. 10.1121/1.389016 6841817

[bibr17-23312165241240353] Moore B. C. J. (2020). Diagnosis and quantification of military noise-induced hearing loss. The Journal of the Acoustical Society of America, 148, 884–894. 10.1121/10.0001789 32873002

[bibr18-23312165241240353] Moore B. C. J. Humes L. E. Cox G. Lowe D. A. Gockel H. E. (2022b). Modification of a method for diagnosing noise-induced hearing loss sustained during military service. Trends in Hearing, 26, 1–9. 10.1177/23312165221145005 PMC9761234 36518073

[bibr19-23312165241240353] Moore B. C. J. Lowe D. A. Cox G. (2022a). Guidelines for diagnosing and quantifying noise-induced hearing loss. Trends in Hearing, 26, 1–21. 10.1177/23312165221093156 PMC9052822 35469496

[bibr20-23312165241240353] Moore B. C. J. Schlittenlacher J. (2023). Diagnosing noise-induced hearing loss sustained during military service using deep neural networks. Trends in Hearing, 27, 1–9. 10.1177/23312165231184982 PMC10408324 37550005

[bibr21-23312165241240353] Moore B. C. J. von Gablenz P. (2021). Sensitivity and specificity of a method for diagnosis of military noise-induced hearing loss. The Journal of the Acoustical Society of America, 149, 62–65. 10.1121/10.0002977 33514161

[bibr22-23312165241240353] Murphy W. J. Fackler C. J. Berger E. H. Shaw P. B. Stergar M. (2015). Measurement of impulse peak insertion loss from two acoustic test fixtures and four hearing protector conditions with an acoustic shock tube. Noise & Health, 17, 364–373. 10.4103/1463-1741.165067 26356380 PMC4617327

[bibr23-23312165241240353] Neitzel R. Seixas N. (2005). The effectiveness of hearing protection among construction workers. Journal of Occupational and Environmental Hygiene, 2, 227–238. 10.1080/15459620590932154 15788384

[bibr24-23312165241240353] Niskar A. S. Kieszak S. M. Holmes A. E. Esteban E. Rubin C. Brody D. J. (2001). Estimated prevalence of noise-induced hearing threshold shifts among children 6 to 19 years of age: The third national health and nutrition examination survey, 1988-1994, United States. Pediatrics, 108, 40–43. 10.1542/peds.108.1.40 11433052

[bibr25-23312165241240353] Passchier-Vermeer W. (1974). Hearing loss due to continuous exposure to steady-state broad-band noise. The Journal of the Acoustical Society of America, 56, 1585–1593. 10.1121/1.1903482 4427029

[bibr26-23312165241240353] Pettersson H. Burstrom L. Hagberg M. Lundstrom R. Nilsson T. (2012). Noise and hand-arm vibration exposure in relation to the risk of hearing loss. Noise & Health, 14(59), 159–165. 10.4103/1463-1741.99887 22918146

[bibr27-23312165241240353] Phillips S. L. Henrich V. C. Mace S. T. (2010). Prevalence of noise-induced hearing loss in student musicians. International Journal of Audiology, 49, 309–316. 10.3109/14992020903470809 20233141

[bibr28-23312165241240353] Pudrith C. Phillips S. Labban J. (2022). Association of self-reported noise exposure and audiograms processed with algorithms proposed to quantify noise-induced hearing loss. International Journal of Audiology, 61, 809–817. 10.1080/14992027.2021.1983216 34634215

[bibr29-23312165241240353] Rosowski J. J. (1991). The effects of external- and middle-ear filtering on auditory threshold and noise-induced hearing loss. The Journal of the Acoustical Society of America, 90, 124–135. 10.1121/1.401306 1880280

[bibr30-23312165241240353] Schust M. (2004). Effects of low frequency noise up to 100 Hz. Noise & Health, 6, 73–85. 15273025

[bibr31-23312165241240353] Smoorenburg G. F. (1992). Speech reception in quiet and in noisy conditions by individuals with noise-induced hearing loss in relation to their tone audiogram. The Journal of the Acoustical Society of America, 91, 421–437. 10.1121/1.402729 1737889

[bibr32-23312165241240353] Turcot A. Girard S. A. Courteau M. Baril J. Larocque R. (2015). Noise-induced hearing loss and combined noise and vibration exposure. Occupational Medicine (London), 65, 238–244. 10.1093/occmed/kqu214 25759070

